# New classification-based global optimization approach for sustainable active power distribution networks

**DOI:** 10.1038/s41598-026-48973-7

**Published:** 2026-04-28

**Authors:** Rasha Elazab, Abdelazim Salem

**Affiliations:** 1 Faculty of Engineering, Capital University, (Formerly: Helwan University), Cairo, Egypt; 2 College of engineering, October 6 University, Giza, Egypt

**Keywords:** Active Power Distribution Networks, Distributed Generation (DG), Capacitor Bank (CB), Voltage Stability, Power Loss Minimization, Radial Distribution Systems, Sustainable Development Goals (SDG), Sustainable Power Systems, Engineering, Electrical and electronic engineering

## Abstract

Active power distribution networks are evolving with the integration of distributed energy resources (DERs) and advanced optimization techniques to enhance grid flexibility and efficiency. However, radial distribution networks suffer from significant voltage drops and high-power losses due to their inherent topology and unidirectional power flow.This study proposes a novel Classification-based Global Optimization (CGO) approach that integrates electrical engineering principles with a structured optimization framework—a departure from conventional metaheuristic methods. Unlike black-box algorithms, CGO classifies distribution buses based on voltage sensitivity and power flow characteristics before applying a deterministic global optimization function for optimal placement and sizing of distributed generation (DG) and capacitor banks (CBs). The methodology is validated on IEEE 33-bus and IEEE 69-bus test systems. For the IEEE 33-bus system, simultaneous DG and CB integration achieved a 94.75% reduction in active power losses, while for the IEEE 69-bus system, losses were reduced by 98.061%. Voltage stability was significantly improved, with the voltage stability index (VSI) increasing to 0.9740 and 0.9773 for the 33-bus and 69-bus systems, respectively. The proposed CGO approach demonstrates superior computational efficiency, with average simulation times of 18.62 s (33-bus) and 21.45 s (69-bus) for combined DG and CB optimization. By enhancing energy efficiency and renewable integration, the method directly supports Sustainable Development Goals (SDGs) 7, 9, 11, and 13, offering a scalable and interpretable solution for modern active distribution networks.

**Supplementary Information:**

The online version contains supplementary material available at 10.1038/s41598-026-48973-7.

## Introduction

Active power distribution systems, characterized by the integration of distributed energy resources (DERs) and advanced control mechanisms, play a pivotal role in modernizing electrical networks. These systems enhance grid flexibility, improve energy efficiency, and enable the incorporation of renewable energy sources, contributing significantly to the achievement of Sustainable Development Goals (SDGs). For instance, integrating electric vehicles (EVs) with virtual power plants (VPPs) can enhance grid resilience and mitigate energy losses during natural disasters, supporting SDG 7 (Affordable and Clean Energy) and SDG 13 (Climate Action)^1^. Additionally, optimizing large-scale urban distribution networks, considering the regulation capacity of the main network, maximizes the consumption of distributed photovoltaics and minimizes power fluctuations, aligning with SDG 11 (Sustainable Cities and Communities)^2^. Furthermore, the transition towards smart grids highlights the importance of sustainable energy sources and renewables integration, directly contributing to SDG 9 (Industry, Innovation, and Infrastructure). Together, these advancements in active power distribution systems not only bolster grid performance but also promote sustainable development across multiple dimensions.

Despite their advantages, radial distribution systems face significant challenges such as voltage drops and high-power losses, stemming from their inherent design and operational characteristics. The radial configuration, combined with increasing load demands, leads to substantial voltage drops and power losses, which reduce system efficiency and reliability. Recent advancements in power system optimization have focused on enhancing the integration and management of DERs.

Recent research has explored advanced optimization strategies for integrating distributed generators (DGs) into modern power systems, with a focus on enhancing technical performance, reliability, and planning efficiency. In^3^, a multi-objective firefly algorithm combined with fuzzy decision-making was proposed to optimally allocate DGs, demonstrating significant improvements in voltage profiles and loss minimization compared to conventional methods. Building on these insights^4^, introduced a fuzzified firefly optimization approach that notably improved grid resilience and efficiency, especially under varying load and generation scenarios. Similarly^5^, applied fuzzy firefly optimization to enhance distribution system pliability and planning, showing its effectiveness in accommodating future load growth and renewable integration. Finally^6^, developed a multi-objective fuzzy spark firefly optimization algorithm tailored for harnessing off-grid power sources, which improved grid voltage stability and overall reliability in isolated and weakly interconnected networks. Collectively, these studies demonstrate that fuzzy-enhanced firefly optimization techniques can serve as robust tools for achieving resilient, efficient, and adaptable DG integration in evolving power systems.

To address these challenges, several studies have focused on the optimal integration of Capacitor Banks (CBs) and distributed generation (DG) in radial distribution systems to minimize power losses and improve voltage stability. Kowsalya and Mohamed Imran^7^ investigated the joint placement of DG and CBs using an optimization-based approach, aiming to reduce power losses and improve overall system efficiency. El-Fergany and Abdelaziz^8^ employed the Cuckoo Search Algorithm (CSA) to optimize CB placement, achieving significant improvements in network performance. Pamuk and Uzun^9^ utilized the Arithmetic Optimization Algorithm (AOA) for efficient DG and CB allocation, demonstrating its effectiveness in loss minimization. Balu and Mukherjee^10^ proposed the Student Psychology-Based Optimization Algorithm (SPBOA) to optimize DG placement, highlighting its potential for enhancing distribution system performance. Mustapha et al.^11^ examined the impact of combined DG and D-STATCOM allocation under various loading conditions, emphasizing the importance of coordinated optimization strategies in active distribution networks. These studies underscore the substantial role of advanced optimization techniques in improving power distribution system efficiency through the integration of CBs and DG.

Further research has explored the optimal allocation of DG and CB placement in radial distribution systems to improve overall system performance. Kayal et al.^12^ introduced an analytical method for DG placement and sizing to enhance voltage stability and reduce power losses. Imran et al.^13^ proposed a novel technique combining network reconfiguration with DG placement for optimized power distribution. Nagaballi and Kale^14^ applied Pareto optimality and game theory to determine the optimal deployment of DG, focusing on maximizing techno-economic benefits. Pham et al.^15^ utilized the Enhanced Coyote Optimization Algorithm (ECOA) to identify the best DG locations and sizes. Sharma et al.^16^ developed a Quasi-Oppositional Swine Influenza Model-Based Optimization (QOSIMBO) with quarantine to improve DG allocation. Balu and Mukherjee^17^ applied SPBOA for optimal DG siting and sizing. Shuaib et al.^18^ addressed CB placement using the Gravitational Search Algorithm (GSA) to improve power quality and reduce losses. These studies collectively demonstrate the effectiveness of various optimization techniques in enhancing the efficiency and reliability of radial distribution systems.

In addition, Ehsanbakhsh and Sepasian^19^ investigated the simultaneous siting and sizing of Soft Open Points (SOPs) and the allocation of tie switches in active distribution networks, incorporating network reconfiguration to improve system flexibility and efficiency. Gil-González et al.^20^ optimized the selection and placement of fixed-step CBs using a discrete version of the Vortex Search Algorithm (DVSA), improving voltage profiles and reducing power losses. These studies further emphasize the role of advanced optimization techniques in enhancing the performance and reliability of modern distribution systems.

Although heuristic and metaheuristic optimization methods have shown promising results in addressing these issues, they are often limited by key challenges. These techniques are highly dependent on tuning hyperparameters, lack robustness across different network topologies, and may converge with local optima without considering fundamental electrical engineering principles. Metaheuristic algorithms operate as black-box approaches, making it difficult to interpret their optimization decisions from an electrical engineering perspective. Moreover, their computational complexity increases significantly as the network size grows, limiting their applicability for large-scale distribution networks.

To address these challenges, this study proposes a Classification-based Global Optimization (CGO) approach, which integrates electrical engineering fundamentals with a structured optimization framework. The key contributions of this study are as follows:

Engineering-Based Classification of Buses: Unlike purely heuristic approaches, the proposed method classifies distribution network buses based on electrical parameters such as voltage sensitivity, power flow, and network topology. This classification ensures that optimization is guided by an engineering-based rationale rather than relying solely on algorithmic randomness. Main contributions of this study can be summarized as follows:

- **Novel Optimization Approach**: A classification-based global optimization (CGO) method is proposed, integrating engineering-based classification with global optimization to enhance power distribution network performance.
- **Improved Voltage Stability & Loss Reduction**: The proposed method achieves 35.62% and 69.13% power loss reduction for capacitor bank (CB) and distributed generation (DG) integration, respectively, in the IEEE 69-bus system, and 98.06% when both are combined.
- **Engineering-Based Optimization vs. Metaheuristics**: Unlike traditional metaheuristic algorithms, the proposed method incorporates electrical engineering principles, improving solution reliability and interpretability while reducing computational complexity.
- **Sustainability & SDG Alignment**: The study supports SDG 7 (Affordable and Clean Energy), SDG 9 (Industry, Innovation, and Infrastructure), and SDG 11 (Sustainable Cities and Communities) by enhancing energy efficiency and integrating renewable energy sources in active distribution networks.
- **Scalability**: The method is tested on IEEE 33-bus and 69-bus systems, demonstrating its applicability for real-world power distribution networks with significant improvements in grid flexibility and resilience.

The remainder of this paper is as follows: Sect.  2 presents the detailed methodology of the proposed Classification-based Global Optimization (CGO) approach. Section  3 provides features of the studied cases. Section  4 discusses the findings, emphasizing the advantages of CGO results and a comparative analysis with existing optimization techniques. Finally, Sect.  5 concludes the paper with key insights and future research directions.

By integrating engineering domain knowledge with a structured optimization framework, the proposed CGO approach bridges the gap between conventional metaheuristic techniques and practical power system optimization, ensuring effective and interpretable solutions for active power distribution networks.

## Methodology

This study introduces a structured optimization framework for the optimal placement and sizing of DGs and CBs in APDNs. Unlike metaheuristic-based approaches, this method employs bus classification followed by a global optimization function, minimizing power losses and improving the voltage profile without relying on iterative stochastic algorithms.

### Objective functions and constraints

The optimization problem consists of two primary objectives:

- *Voltage Profile Improvement*.

The objective function ensures that the bus voltages remain within the permissible limits while minimizing deviations from the nominal values:

1 $$\:{\mathrm{F}}_{\mathrm{V}}=\sum\:_{\mathrm{i}=1}^{\mathrm{N}}{(1-\left|{\mathrm{V}}_{\mathrm{i}}\right|)}^{2}$$

*Where*, $\:\left|{V}_{i}\right|$: *is the voltage magnitude at bus number i*,* with proper voltage regulation constraints.*

- *Reduction of Losses*.

1-Active power loss objective function:

2 $$\:{\mathrm{F}}_{\mathrm{p}}=\sum\:_{\mathrm{i}=1}^{\mathrm{N}\mathrm{s}}{I}_{si}^{2}\:\times\:\:{\mathrm{R}}_{\mathrm{s}\mathrm{i}\:\:\:\:}$$

2- Reactive power loss objective function:

3 $$\:{\mathrm{F}}_{\mathrm{q}}=\sum\:_{\mathrm{i}=1}^{\mathrm{N}\mathrm{s}}{I}_{si}^{2}\:\times\:\:{\mathrm{X}}_{\mathrm{s}\mathrm{i}}$$

*where*: $\:{I}_{si}$ : *is the current magnitude in i*^*th*^ *section*,* and* $\:{R}_{si}$, $\:{X}_{si}$ : *are the series resistance and reactance of i*^*th*^ *section*,* respectively.*

3- Power flow constraints:

Before integrating DGs and CBs:

4 $$\:{\mathrm{P}}_{\mathrm{S}\mathrm{l}\mathrm{a}\mathrm{c}\mathrm{k}}=\sum\:_{\mathrm{i}=1}^{\mathrm{N}}{\mathrm{P}}_{\mathrm{l}\mathrm{d},\mathrm{i}}+{\mathrm{P}}_{\mathrm{l}\mathrm{o}\mathrm{s}\mathrm{s}}$$

5 $$\:{\mathrm{Q}}_{\mathrm{S}\mathrm{l}\mathrm{a}\mathrm{c}\mathrm{k}}=\sum\:_{\mathrm{i}=1}^{\mathrm{N}}{\mathrm{Q}}_{\mathrm{l}\mathrm{d},\mathrm{i}}+{\mathrm{Q}}_{\mathrm{l}\mathrm{o}\mathrm{s}\mathrm{s}}$$

After integrating DGs and CBs:

6 $$\:{\mathrm{P}}_{\mathrm{S}\mathrm{l}\mathrm{a}\mathrm{c}\mathrm{k}}+\sum\:{\mathrm{P}}_{\mathrm{D}\mathrm{G}\mathrm{s}}=\sum\:_{\mathrm{i}=1}^{\mathrm{N}}{\mathrm{P}}_{\mathrm{l}\mathrm{d}}+{\mathrm{P}}_{\mathrm{l}\mathrm{o}\mathrm{s}\mathrm{s}}$$

7 $$\:{\mathrm{Q}}_{\mathrm{S}\mathrm{l}\mathrm{a}\mathrm{c}\mathrm{k}}=\sum\:_{\mathrm{i}=1}^{\mathrm{N}}{\mathrm{Q}}_{\mathrm{l}\mathrm{d}}+{\mathrm{Q}}_{\mathrm{l}\mathrm{o}\mathrm{s}\mathrm{s}}-\sum\:_{\mathrm{i}=1}^{\mathrm{m}}{\mathrm{Q}}_{{\mathrm{c}}_{\mathrm{i}}}$$

4- Global optimization function.

The overall optimization problem is formulated as a weighted sum of the objective functions:

8 $$\:{\mathrm{F}}_{\mathrm{g}}={\upalpha\:}\mathrm{*}{\mathrm{F}}_{\mathrm{V}}+{\upbeta\:}\mathrm{*}{(\mathrm{F}}_{\mathrm{p}}+{\mathrm{F}}_{\mathrm{q}})$$

*where*: $\:\alpha\:\:,\beta\:\::\:$*Weighting factors ensuring balanced optimization.*

### Optimization framework

Most APDNs supply inductive loads that consume both active and reactive power from the main substation connected at the slack bus bar, these powers flow through the distribution sections from slack bus bar to the network loads, which lead to voltage drop and power losses in these sections. By optimal selection of location and size of DGs and CBs connected to the APDN, the power flow in the distribution sections are reduced, consequentially the flow currents in these sections are minimized, also the voltage drop, and power losses on the distribution sections are reduced. The framework consists of two key stages:

1. Bus classification:

The buses are categorized based on their voltage sensitivity, active power demand, and reactive power demand, ensuring a structured and efficient search space for optimization.

Global optimization function:

The classified buses are evaluated using the global function to determine the best locations and 2. sizes for DGs and CBs.

**Fig. 1:**
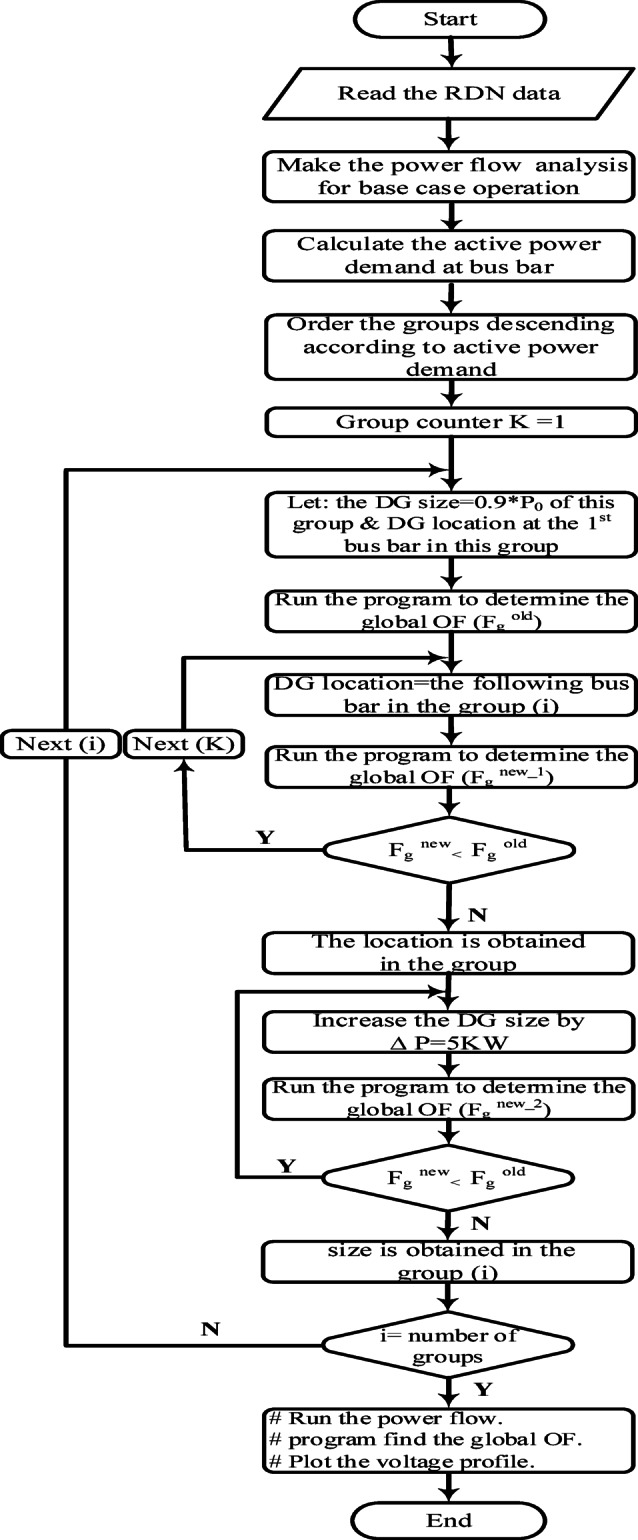
Flowchart for DGs integration.

**Fig. 2:**
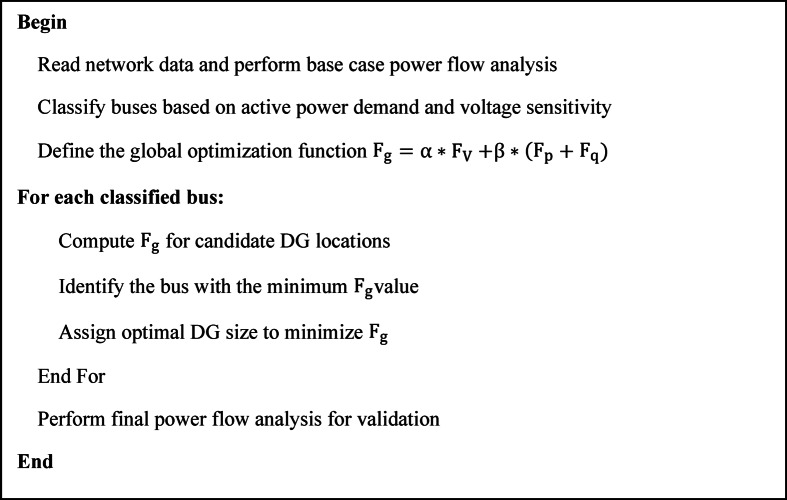
Pseudocode of optimal placement and sizing of DGs.

For DGs integration, the process begins with load flow analysis, ranking buses based on active power demand, and initializing group-wise DG allocation. Global optimization function determines optimal DG locations and sizes, ensuring improved voltage stability and minimized losses. The final configuration is validated through power flow analysis, as shown in Figs. 1 and 2.

**Fig. 3:**
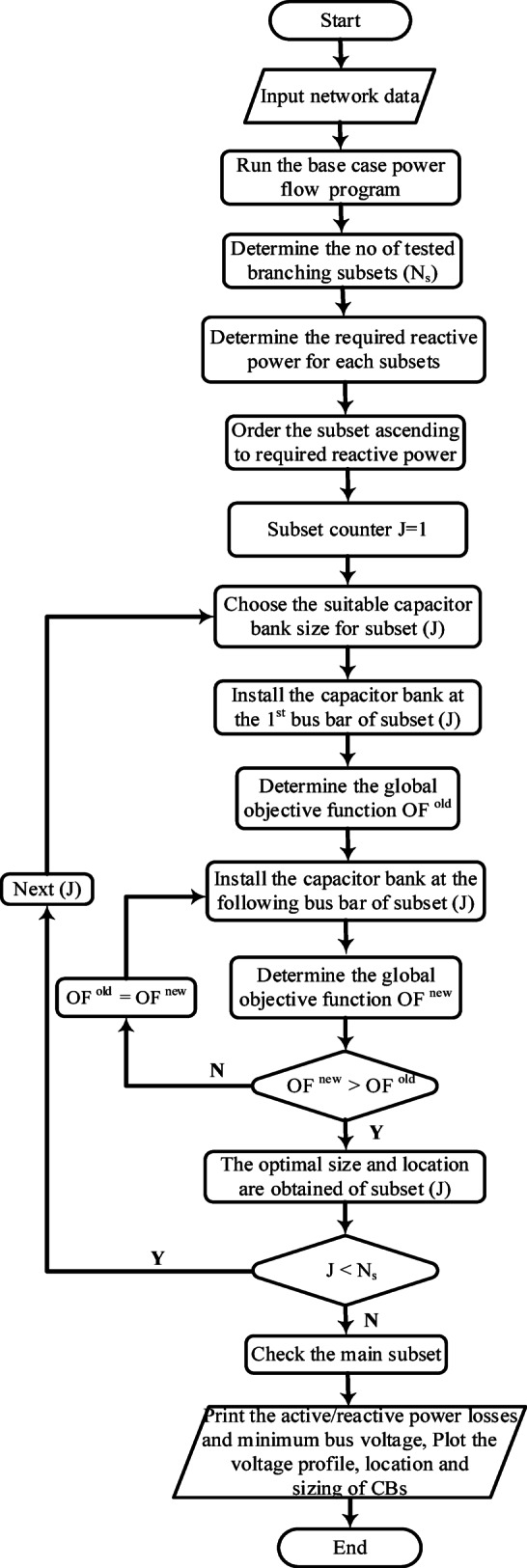
Flowchart for CBs integration.

**Fig. 4:**
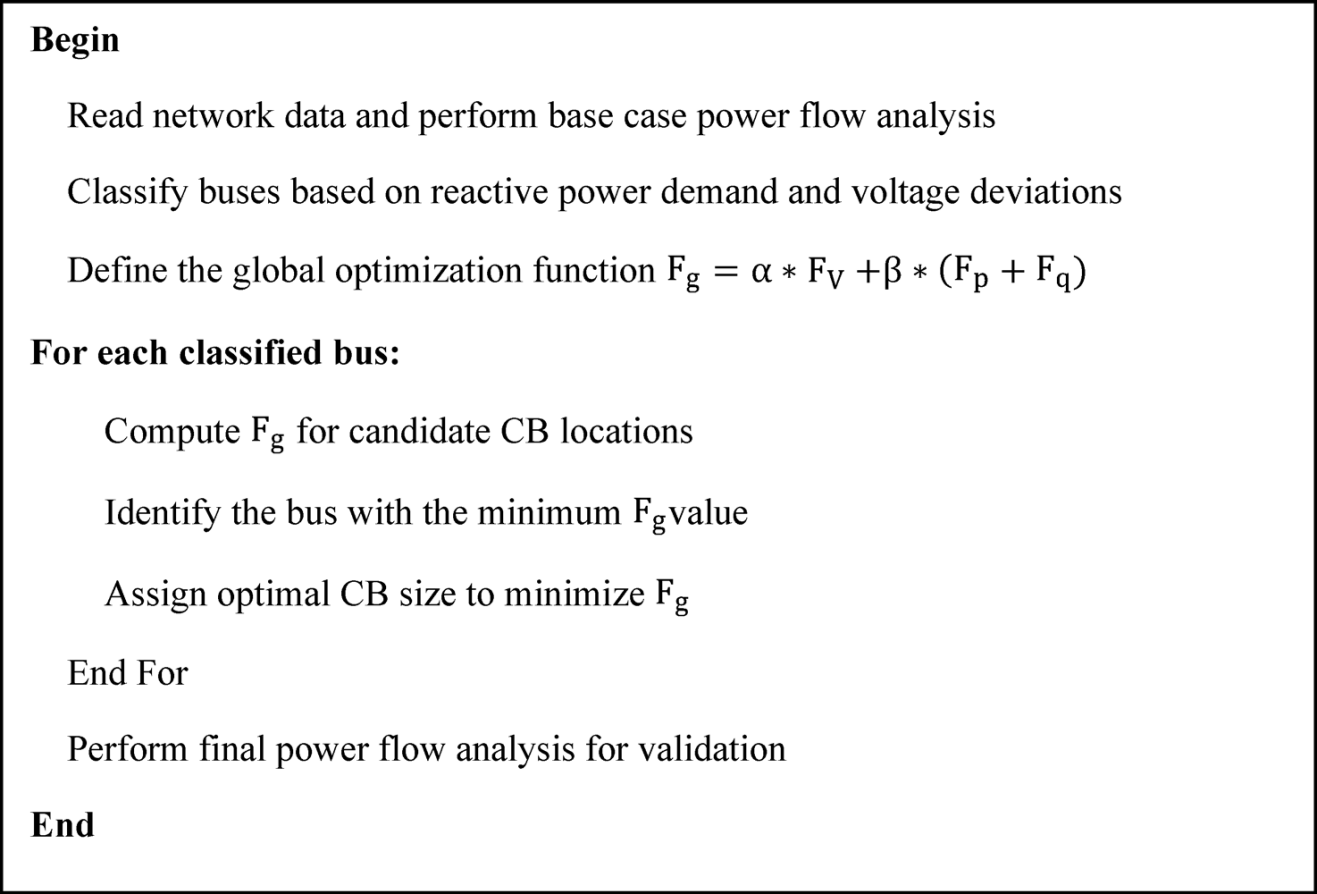
Pseudocode of optimal placement and sizing of CBs.

Buses are classified based on reactive power demand and voltage deviations. The global optimization function is then used to determine the best CB locations and sizes, ensuring voltage stability and reactive power loss minimization, as shown in Figs. 3 and 4.

The proposed methodology offers significant advancement over conventional metaheuristic-based optimization techniques by introducing a structured bus classification approach prior to optimization. The existing methods rely on stochastic search mechanisms, which often suffer from high computational costs, uncertain convergence, and sensitivity to parameter tuning. These methods require extensive iterations to explore a vast solution space, leading to prolonged computation times and potential local optima entrapment. Additionally, their performance varies based on heuristic parameter settings, making them less predictable in large-scale distribution networks.

In contrast, the proposed engineering-based pre-classification method systematically identifies and prioritizes candidate buses before applying a deterministic global optimization function. This structured approach eliminates the need for random exploration, ensuring a more efficient, reliable, and scalable solution. By significantly reducing the search space, it accelerates convergence, minimizes computational complexity, and guarantees robust results without the risk of premature convergence or excessive iteration overhead. Moreover, by leveraging a composite global optimization function that balances voltage profile improvement and power loss minimization, the method enhances overall grid performance with higher precision. The result is an optimized DG and CB placement strategy that is not only computationally efficient but also inherently stable and reproducible, making it a practical choice for real-world power distribution systems.

### Case study

To validate the effectiveness of the proposed methodology, simulations were conducted on two widely recognized benchmark distribution networks: the IEEE 33-bus system and the IEEE 69-bus system. These systems provide a robust testing environment for evaluating the optimal placement and sizing of DGs and CBs while ensuring improved voltage stability and power loss reduction.

A- *IEEE 33-Bus*.

**Fig. 5:**
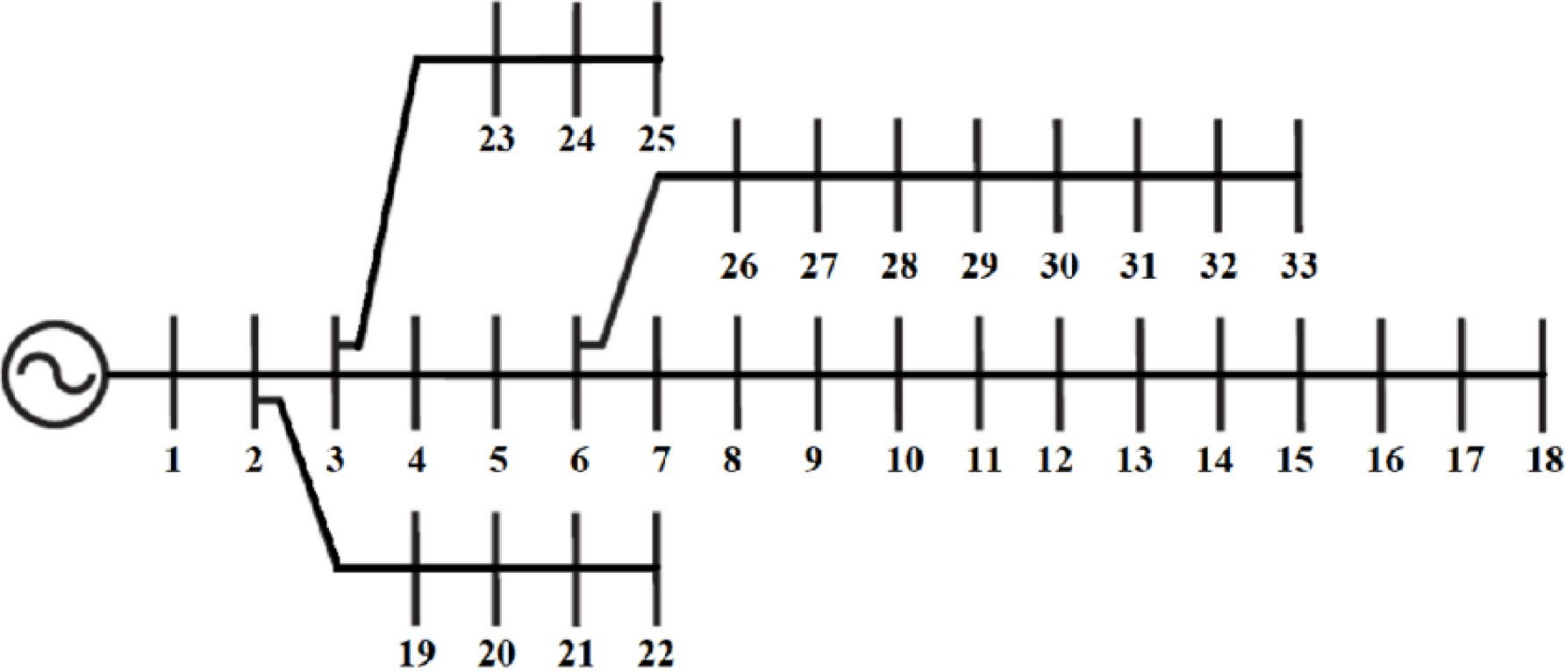
IEEE 33-bus^9^.

The IEEE 33-bus radial distribution network consists of 33 buses and 32 distribution sections, operating at a base voltage of 12.66 kV and a base power of 10MVA, see Fig. 5. The network topology, line impedances, and load demand data are obtained from^9^, with its single-line diagram illustrated in Fig. 5. Under the base case operating condition, the system exhibits active power losses of 210.98 kW and reactive power losses of 143.14kVAR. These losses significantly impact the network’s overall efficiency, necessitating optimal DG and CB placement to enhance voltage regulation and minimize power dissipation.

### IEEE 69-Bus

**Fig. 6:**
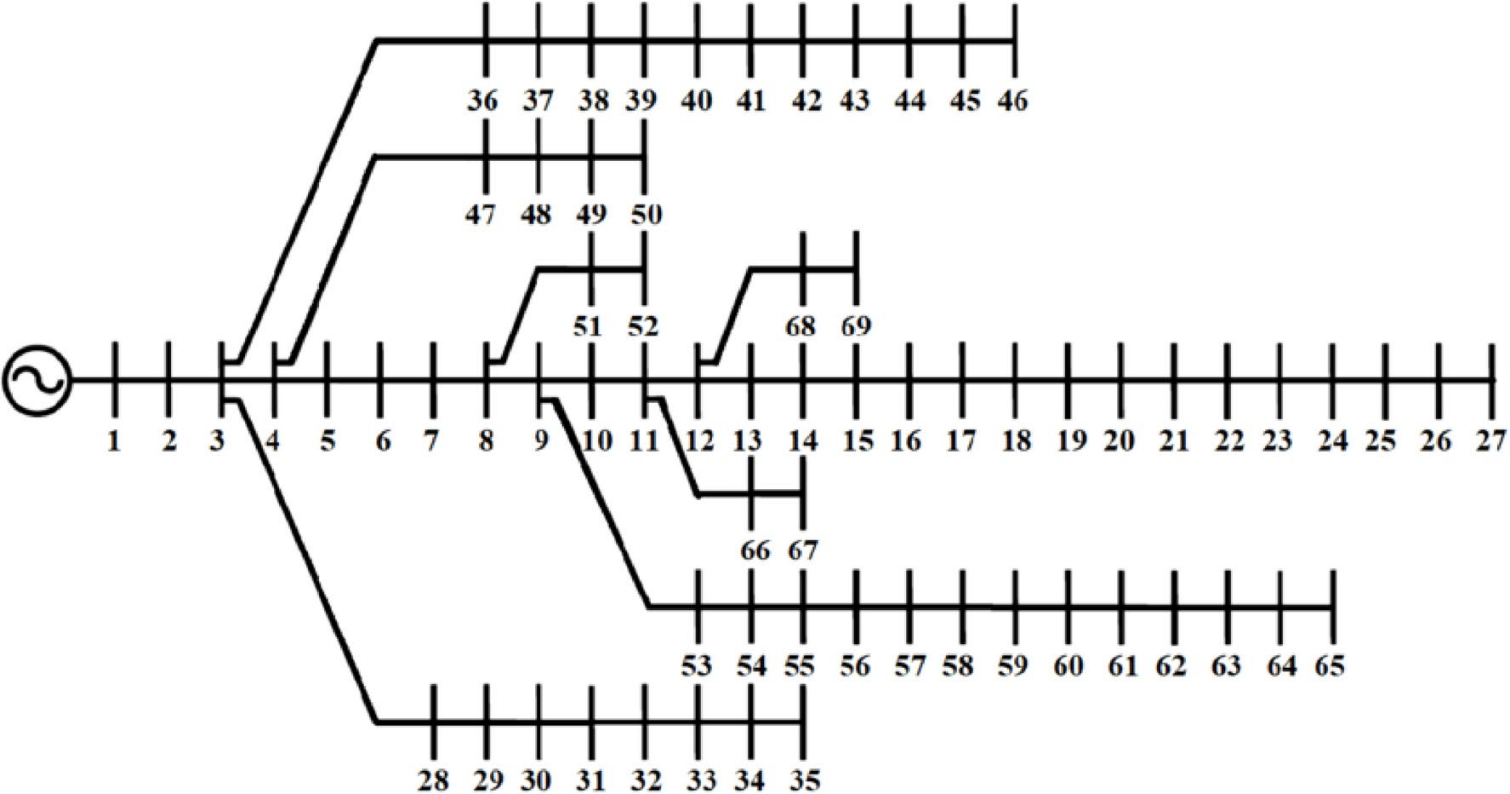
IEEE 69-bus^9^.

The IEEE 69-bus distribution network, sharing the same base voltage (12.66 kV) and base power (10MVA), comprises 69 buses and 68 distribution sections. The system topology, along with its corresponding section and load data [9], see Fig. 6. In its base configuration, the network experiences active power losses of 225.0 kW and reactive power losses of 102.13 kVAR. Given its larger network size, this system presents additional challenges in terms of voltage stability and loss minimization, making it an ideal test case for evaluating the proposed methodology’s scalability and robustness.

Both test systems serve as benchmarks to assess the efficacy of the bus classification-based optimization approach in comparison to various metaheuristic-based studies. By implementing the proposed DG and CB allocation strategy, substantial improvements in voltage profiles and power loss reduction are expected, reinforcing the method’s practical applicability in modern distribution networks.

In this study, DG units are modeled as inverter-interfaced photovoltaic (PV) systems, which convert the DC power generated by solar panels into three-phase AC power via inverters equipped with harmonic filters to ensure acceptable power quality. The inverters are assumed to operate at unity power factor, thereby injecting only active power into the distribution network.

The efficiency of a solar PV panel typically ranges between 15% and 23% in current commercial technologies. A higher efficiency implies greater electrical power generation from the same panel area, which can reduce grid dependence and enhance the technical, economic, and environmental benefits of the system. Conversely, lower efficiency results in reduced energy yield, potentially necessitating a larger PV array to meet the same load demand. Although the present analysis assumes peak irradiance conditions to maintain consistency with conventional steady-state optimization frameworks, it is acknowledged that PV output varies with weather and solar patterns. Future work will incorporate time-series solar irradiance data and dynamic load profiles to better capture real-world operational scenarios.

## Results and discussion

The effectiveness of the proposed bus Classification-based Global Optimization approach (CGO) is demonstrated through simulations. The results are analyzed for three operational scenarios: (1) the installation of CBs only, (2) the installation of DGs only, and (3) the simultaneous installation of both CBs and DGs. The results obtained are benchmarked against multiple existing optimization techniques to highlight the superior performance of the proposed approach.

### IEEE 33 bus

#### CBs integration only

In this scenario, the optimal placement and sizing of CBs were determined using the CGO algorithm. The results, presented in Table 3, show that the optimal CB capacities are 450kVAR, 300kVAR, 450kVAR, and 900kVAR, installed at buses 6, 13, 24, and 30, respectively. This configuration led to total active and reactive power losses of 136.987 kW and 93.5351kVAR respectively, representing a 35.071% and 34.65% reduction compared to the base case, while the minimum voltage becomes 0.9309 p.u. that is increased by 3% related to the base case voltage. A comparative analysis with other published methods indicates that the proposed approach outperforms existing techniques by achieving a lower power loss, as summarized in Table 1.

**Table 1:** IEEE 33-bus system results for CB-only.

Method	Bus No.	CBs capacities (kVAR)	Power Loss (kW)	Loss Reduction (%)	Simulation time (sec)
IP^7^	9, 29, 30	450, 800, 900	171.78	18.58	N/A
SA^7^	10, 30, 14	450, 350, 900	151.75	28.07	N/A
BFOA^8^	18, 30, 33	349.6, 820.6, 277.3	144.04	31.72	N/A
AOA^9^	8, 24, 30	556, 513, 965	139.41	33.92	N/A
CGO	6, 13,24,30	450,300,450,900	136.987	35.071	12.5

#### DGs integration only

The optimal placement and sizing of DGs operating at unity power factors were also determined using the CGO algorithm. The results, detailed in Table 2, indicate that DGs were installed on buses 14, 30, and 24, with capacities of 770 kW, 1065 kW, and 1097 kW, respectively. This installation significantly reduced the active and reactive power losses from 210.98 kW and 143.1283kVAR in the base case to 72.789 kW and 50.7193kVAR, corresponding to a 65.51% and 64.563% reduction, but the minimum bus voltage has a value of 0.9686 p.u. that represents an incremental in it by 7.17%. Comparing the obtained results with other published optimization techniques, the proposed method demonstrates competitive performance, achieving one of the lowest active power losses. Table 2 summarizes the comparative results.

**Table 2:** IEEE 33-bus system results for DG-only.

Method	Optimal Location (Bus No.)	Optimal DG Size (MW)	Power Loss (kW)	Loss Reduction (%)	Simulation time (sec)
AM^12^	13, 29, 31	1.121, 1.027, 0.126	89.5	57.28	N/A
FWA^13^	14, 18, 32	0.5897, 0.1895, 1.0146	88.68	56.24	N/A
MTLBO^14^	23, 32, 15	1.066, 0.847, 0.885	80.22	62	N/A
JAYA^14^	29, 25, 12	0.921, 0.795, 1.11	76.66	63.6	N/A
COA^15^	14, 25, 30	0.7096, 0.5954, 0.9972	76	63.98	N/A
ECOA^15^	14, 25, 30	0.7376, 0.6518, 1.0708	74.6	64.64	N/A
SIMBO-Q^16^	14, 24, 29	0.7438, 1.0415, 1.1352	73.4	65.20	N/A
QOSIMBO-Q^16^	14, 24, 30	0.7708, 1.0965, 1.0655	72.8	65.48	N/A
AA^10^	13, 24, 30	0.7, 1.017, 1.012	72.89	65.45	N/A
HHO^18^	13, 24, 30	0.8173, 1.0829, 1.0465	72.80	65.496	N/A
SPBO^18^	14, 24, 30	0.7723, 1.1059, 1.0658	72.79	65.5003	N/A
AOA^9^	14, 24, 30	0.7764, 1.0999, 1.0702	72.79	65.50	N/A
CGO	14,30,24	0.770,1.065,1.097	72.789	65.51	12.6

#### Both CBs & DGs integration

When both CBs and DGs were optimally installed using the proposed algorithm, the results obtained indicate a substantial improvement in power loss reduction. In this scenario, DGs were positioned at buses 14, 24, and 30, with capacities of 770 kW, 1097 kW, and 1065 kW, respectively. Simultaneously, CBs were placed at buses 6, 13, 24, and 30, with respective capacities of 450kVAR, 300kVAR, 450kVAR, and 900kVAR. This combined installation reduced the total active and reactive power losses to 11.0758 kW and 9.4971kVAR, achieving a remarkable 94.75% and 93.364% loss reductions respectively, while the minimum voltage becomes 0.9934 p.u. (with incremental of 9.914%). The proposed method is benchmarked against alternative algorithms in Table 3.

**Table 3:** IEEE 33-bus system results for combined CBs and DGs integration.

Method	Bus No.	DG Size (MW)	Bus No.	CB Size (kVAR)	Power Loss (kW)	Loss Reduction (%)	Simulation time (sec)
GA^21^	16,22,30	0.25,0.25,0.5	18,29,30	300,300,600	71.25	64.661	N/A
BFOA^8^	17,18,33	0.5424,0.1604,0.8955	18,30,33	163.2,541.0, 338.2	41.44	80.37	N/A
DVSA^20^	25,29,11	0.973,1.04,0.563	23,30,14	465,565,535	24.688	87.82	N/A
AOA^9^	14,25,30	0.794,0.881,1.100	14,25,30	384,436,1009	12.6571	94.00	N/A
CGO	14,30,24	770,1065,1097	6,13,24,30	450,300,450,900	11.0758	94.75049	18.62

#### Scenarios Voltage profile

**Fig. 7:**
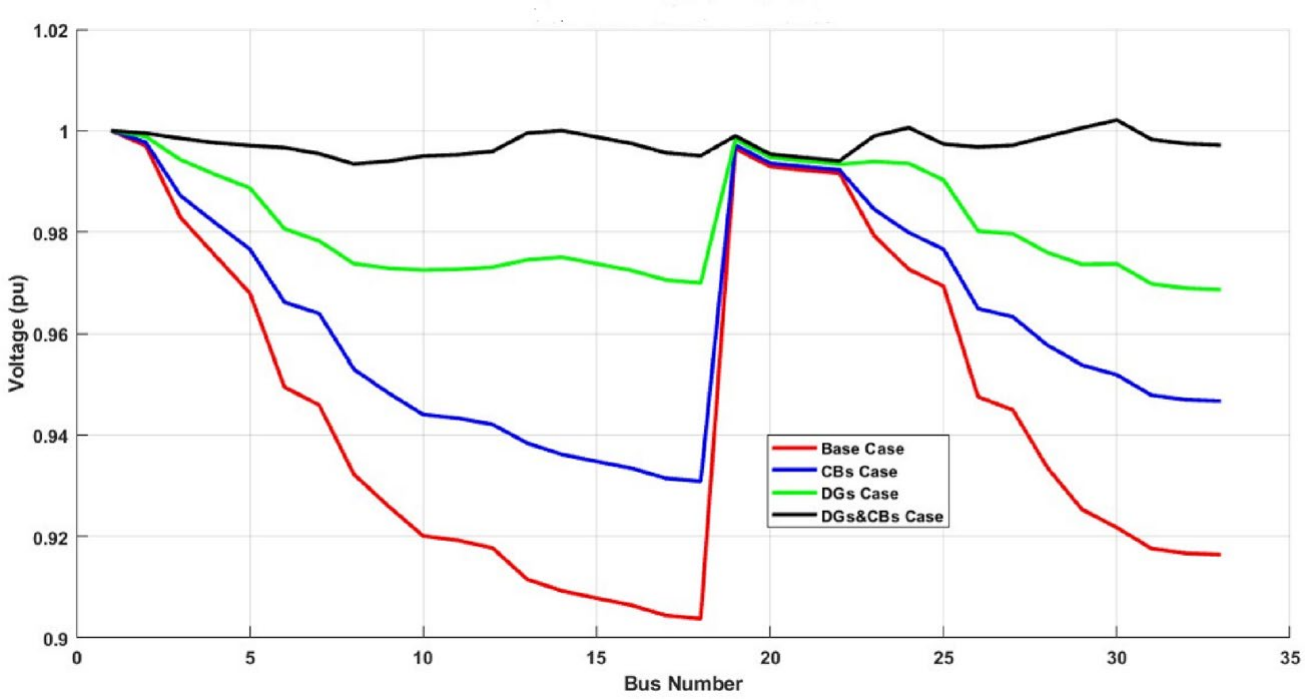
IEEE 33 bus cases voltage profiles.

Figure 7 illustrates the voltage profiles under different operational scenarios. The base case operates with significant voltage drops. The lowest voltage reaches a value of 0.9038 p.u. at bus bar 18, highlighting the need for voltage support. The inclusion of CBs improves the voltage profile by supplying reactive power support, so the minimum bus voltage becomes 0.9309 p.u. at bus bar 18. However, the enhancement remains moderate since CBs do not contribute to active power compensation.

The placement of DGs at optimal locations significantly improves the voltage profile due to active power injection, while the minimum bus voltage becomes 0.9686 p.u. at bus bar 33. The overall voltage levels are notably higher compared to the CBs-only case. Simultaneous CBs & DGs installation yields the best voltage profile, maintaining bus voltages close to 1.0 p.u., where the minimum bus voltage becomes 0.9934 p.u. at bus bar 8. throughout the network. The combined effect of active and reactive power compensation minimizes voltage deviations, ensuring enhanced system stability and power quality.

This comparison highlights that while CBs improve voltage by reducing reactive power demand, the combination of DGs and CBs provides a more comprehensive solution, achieving superior voltage regulation across the network. But for studying the effect of the new proposed technique on the value of voltage stability index for this network, the VSI is changed from 0.6672 at the base case to 0.7509 for the network with connected CBs, while VSI becomes 0.8803 with DG only, but with CBs and DGs it becomes the greatest one of 0.9740. That means the distribution network becomes more stable.

### IEEE 69 bus

#### CBs integration only

The proposed method optimally places CBs at buses 8, 21, 12, and 61, with capacities 340kVAR, 200kVAR, 280kVAR, and 1200kVAR, respectively. This configuration effectively enhances the voltage profile by reducing reactive power demand, mitigating voltage drops, and improving system stability. The total active and reactive power losses are reduced from 225.0 kW to 144.8566 kW and 102.1658kVAR to 67.5597kVAR, achieving 35.6193% and 33.8724% reductions, with minimum voltage of 0.9315 p.u. (the incremental percentage is 2.453%). Compared to other published algorithms, the proposed technique demonstrates superior performance, achieving the highest loss reduction percentage, as shown in Table 4.

**Table 4:** IEEE 69-bus system results for CB-only.

Method	Bus No.	CB Size (kVAR)	Power Loss (kW)	Loss Reduction (%)	Simulation time (sec)
CSA^11^	9,21,61	600,250,1200	147.95	34.21	N/A
SA^7^	58,11,59	900,450,600	155.45	30.91	N/A
GSA^7^	26,13,15	150,150,1050	145.9	35.16	N/A
AA^10^	11,21,61	368,231,1196	145.21	35.46	N/A
AOA^9^	11,21,61	410,233,1234	145.096	35.51	N/A
CGO	8, 21, 12, 61	340,200,280,1200	144.8566	35.6193	14.67

#### DGs integration only

In this case, DGs are optimally placed at buses 21, 61, and 11, with capacities 0.340 MW, 1.732 MW, and 0.560 MW, respectively. DGs provide active power support, significantly enhancing voltage levels and reducing losses. The total active and reactive power losses are reduced to 69.45 kW and 34.9706kVAR, corresponding to 69.133% and 65.771% reductions, but the minimum voltage has a value of 0.9794 p.u. (with 7.721% increasing), as shown in Table 5 the active power loss is the only mentioned in the published works. The proposed placement strategy outperforms several well-established optimization techniques, ensuring an efficient reduction in power losses and voltage deviations.

**Table 5:** IEEE 69-bus system results for DG integration only.

Method	Optimal Location (Bus No.)	Optimal DG Size (MW)	Power Loss (kW)	Loss Reduction (%)	Simulation time (sec)
FWA^13^	65,61,27	0.4085,1.1986,0.2258	77.85	65.39	N/A
MTLBO^14^	20,62,57	0.446,1.8360.477	77.36	65.61	N/A
JAYA^14^	61,50,12	2.00,0.100,1.016	75.83	66.29	N/A
SIMBO-Q^16^	61,9,17	1.500,0.6189,0.5297	71.3	68.3	N/A
AA^10^	11,17,61	0.499,0.377,1.668	69.55	69.09	N/A
SPBO^18^	11,18,61	0.5599,0.3692,1.1731	69.45	69.13	N/A
MFO^7^	11,61,21	0.54917,1.7458,0.35254	69.45	69.13	N/A
AOA^9^	11,61,21	0.5716,1.7199,0.341	69.43	69.13	N/A
CGO	21,61,11	0.340,1.732,0.560	69.45	69.13	14.75

#### Both CBs & DGs integration

When both DGs and CBs are installed simultaneously at their optimal locations, the system experiences the most significant improvements. This approach leverages the complementary benefits of reactive power compensation (CBs) and active power injections (DGs), resulting in near-optimal voltage regulation and minimal power losses. The total active and reactive power losses are reduced to 4.3620 kW and 6.7789kVAR, achieving an impressive 98.061% and 93.365% reductions, while the minimum voltage at this condition becomes of 0.9943 p.u. (that represents 9.36% improvement), the obtained results represent the best among all compared methods, see Table 6. The voltage profile is significantly improved, maintaining bus voltages close to 1.0 p.u., thereby ensuring system reliability and power quality.

In the proposed CGO-based method, the average simulation time for the combined DG and CB integration was approximately **21.45 s**, demonstrating high computational efficiency compared to typical metaheuristic approaches applied to the IEEE 69-bus system.

**Table 6:** IEEE 69-bus system results for combined CBs and DGs integration.

Method	Bus No.	DG Size (MW)	Bus No.	CB Size (kVAR)	Power Loss (kW)	Loss Reduction (%)
WCA^20^	17,61,69	0.5408,2.0, 1.1592	2,62,69	1187.9,1237.3,269.7	33.339	85.18
BSA^21^	19,22,61	0.294,0.219,1.768	7,2,3	450,300,150	7.6047	96.61
AOA^9^	61,11,21	1.6511,0.521,0.334	61,11,21	1282,414,234	4.6243	97.94
CGO	21,61,11	0.340,1.732,0.560	8, 21, 12, 61	340,200,280,1200	4.362	98.061

#### Voltage profile comparison

**Fig. 8:**
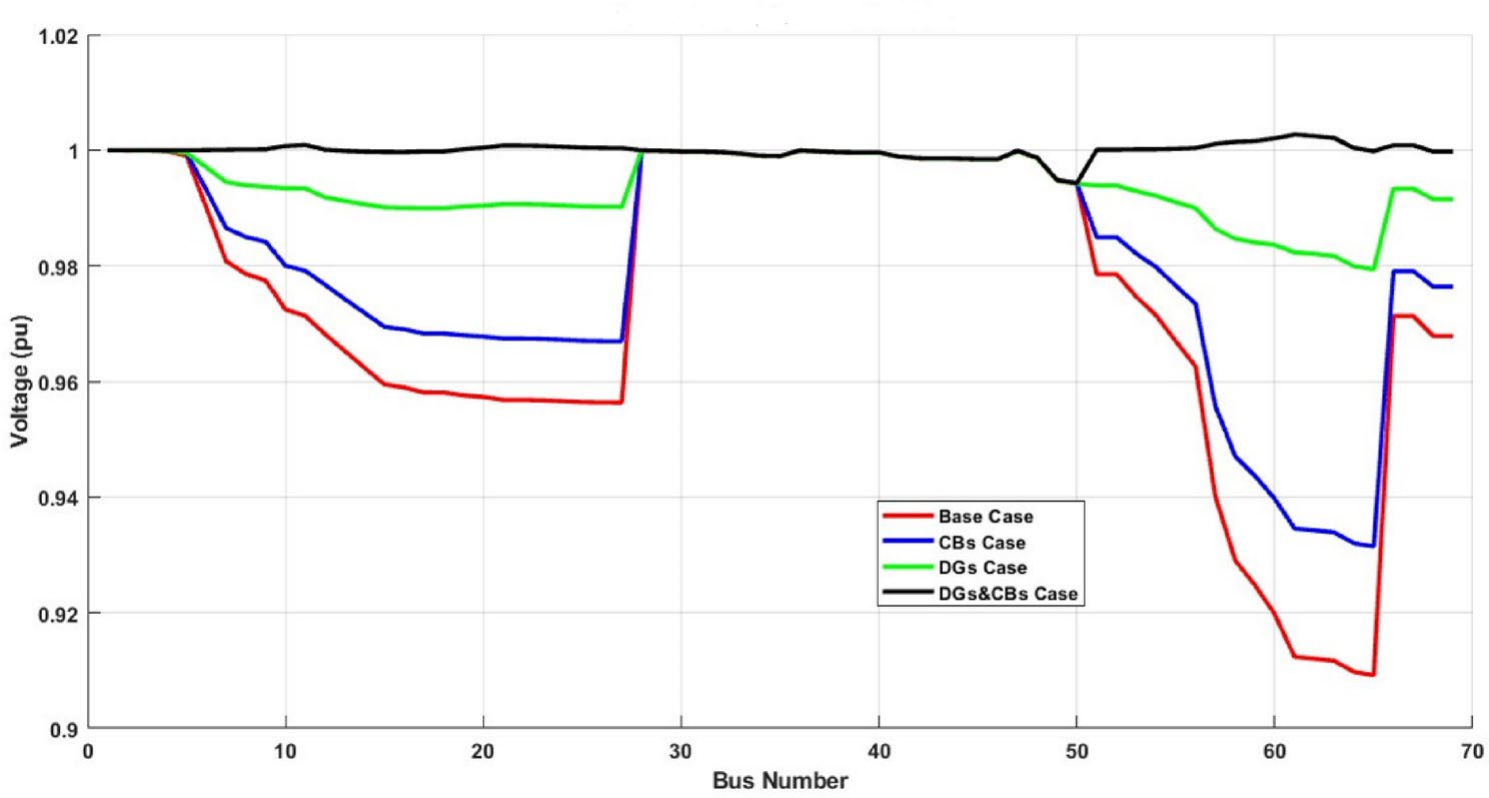
IEEE 69 bus cases voltage profiles.

As shown in Figure 8, the base case suffers from severe voltage drops occur, particularly beyond bus 50, highlighting the need for voltage support, where the minimum bus voltage becomes 0.9092 p.u. at bus bar 65. Voltage levels have improved, but some buses still experience considerable drops at CBs only, while the minimum bus voltage becomes 0.9315 p.u. at bus bar 65. Active power injection from DGs significantly enhances the voltage profile, outperforming the CB-only scenario, so the minimum bus voltage becomes 0.9794 p.u. at bus bar 65. The optimal combination of CBs and DGs provides the best voltage stability, maintaining all buses within an acceptable voltage range, where the minimum bus voltage becomes 0.9943 p.u. at bus bar 50.

For this distribution network, when studying the impact of the proposed algorithm on the value of voltage stability index, the VSI has a value of 0.6833 at the base case, but it is 0.7528 for the network with CBs only, where VSI becomes 0.9201 with DG only, but with CBs and DGs it becomes the greatest one of 0.9773. Which refers to the distribution network becoming more stable.

The results obtained from the proposed classification-based global optimization approach demonstrate its effectiveness in enhancing voltage profiles and minimizing power losses in both the IEEE 33-bus and 69-bus distribution systems. Comparative analysis with recently published techniques confirms that the proposed method outperforms other approaches in both CB placement and DG allocation. For the 33-bus system, the integration of CBs and DGs significantly improved voltage stability while achieving a power loss reduction of 94.75%, demonstrating the effectiveness of the methodology in enhancing overall network performance. Similarly, in the 69-bus system, the proposed approach achieved an impressive 98.061% power loss reduction, highlighting its capability to provide optimal voltage regulation and system efficiency. While the individual installation of CBs or DGs resulted in notable improvements, their simultaneous integration provided the most substantial benefits, ensuring superior power quality, loss minimization, and voltage stability. These findings reinforce the practicality and computational efficiency of the proposed technique, making it a promising solution for real-world distribution system planning and optimization.

While the primary focus of this study is on minimizing power losses and improving voltage profiles, the current-carrying capacity of conductors is indirectly satisfied due to the localized power injection by optimally placed DGs and CBs. These components reduce the burden on upstream feeders by supplying part of the load demand locally, which leads to a general reduction in current magnitudes across the distribution network. However, to enhance the reliability and applicability of the proposed approach, future extensions will incorporate explicit thermal limits of conductors as optimization constraints to ensure conductor ampacity is never exceeded under varying load and generation conditions.

The proposed CGO framework exhibits strong convergence behavior by leveraging a bus classification stage that narrows the search to high-impact locations, thereby reducing the likelihood of stagnation in local minima and guiding the search toward the global optimum with greater consistency. The sensitivity analysis on population size further highlights the importance of balancing exploration and exploitation: smaller populations enable faster convergence but risk premature stagnation, whereas larger populations improve diversity at the expense of runtime; a moderate population size was found to provide the best trade-off between solution quality and computational efficiency. Beyond simulation results, the method is designed for practical deployment in real-world active power distribution networks using readily available SCADA data, allowing utilities to integrate the approach into existing DG and capacitor bank planning workflows for loss minimization and voltage stability enhancement. This combination of robust convergence, tuned parameter selection, and operational feasibility underscores the CGO framework’s potential for both research and industry adoption.

## Conclusion

This study presented a Classification-based Global Optimization (CGO) approach for optimal DG and CB placement in active distribution networks. Unlike metaheuristic methods, CGO integrates engineering-based bus classification with a deterministic global function, offering an interpretable, efficient, and scalable solution.

CGO achieved 94.75% (IEEE 33-bus) and 98.061% (IEEE 69-bus) active loss reduction with combined DG-CB integration, while improving VSI to 0.9740 and 0.9773, respectively. Simulation times averaged 18.62 s (33-bus) and 21.45 s (69-bus), demonstrating computational efficiency superior to conventional metaheuristics.

The work tackled several challenges: (1) accurate modeling of radial networks with inverter-based DGs, (2) multi-objective trade-offs between voltage stability and loss minimization, (3) development of a voltage-sensitivity-based classification heuristic, and (4) scalable validation across different network sizes. By enhancing energy efficiency and renewable integration, the method supports SDGs 7, 9, 11, and 13, promoting sustainable and resilient power systems. Future work will extend CGO to unbalanced networks, time-varying loads, EV integration, and real-world feeder data with operational constraints.

## Supplementary Information

Below is the link to the electronic supplementary material.

Supplementary Material 1

## Data Availability

All data supporting the findings of this study are included in the article and its Supplementary information file.

## Abbreviation

AA: Analytical approach
MFO: Moth flame optimization
AOA: Arithmetic optimization algorithm
MTLBO: Modified teaching learning based optimization
APDN: Active power distribution network
OF^n^: New objective function
BFOA: Bacterial foraging optimization algorithm
OF^o^: Old objective function
BFS: Backward forward sweep method
CBs: Capacitor banks
CGO: Classification-based global optimization
COA: Coyote optimization algorithm
PSO: Particle swarm optimization
CSA: Cuckoo search algorithm
DERs: Distributed energy resources
QOSIMBO: Quasi-oppositional swine influenza model-based optimization
DGs: Distributed generators
DS: Distribution section
SA: Sensitivity analysis
DVSA: Discrete vortex search algorithm
SCADA: Supervisory control and data acquisition
ECOA: Enhanced coyote optimization algorithm
SDG: Sustainable development goals
EV: Electric Vehicle
SIMBO: Swine influenza model-based optimization
Fp: Real power loss objective function
SOP: Soft open point
Fq: Reactive power loss objective function
SPBOA: Student psychology-based optimization algorithm
FV: Voltage level objective function
VPP: Virtual power plant
FWA: Firefly optimization algorithm
VSI: Voltage stability index
GA: Genetic algorithm
WCA: Water cycle algorithm
GSA: Gravitational search algorithm
HHO: Harris hawks optimization
α, β: Weighting factors
JAYA: Jaya algorithm
Vi: i-th Bus Voltage
P_loss_, Q_loss_: Real and reactive power losses
P_ld, i_, Q_ld, i_: Load real and reactive power at bus i
P_slack_: Slack bus real power
Rsi, Xsi: i-th section resistance and reactance
Zsi: i-th section impedance
Isi: i-th section current
Vslack: Slack bus voltage
