# Supplementary material for: New classification-based global optimization approach for sustainable active power distribution networks
Source: Sci Rep. 2026 Apr 28;16:13648. doi: 10.1038/s41598-026-48973-7 (PMC13125413; doi:10.1038/s41598-026-48973-7)
Supplement: Supplementary file 1 — Supplementary Material 1 [file 41598_2026_48973_MOESM1_ESM.docx]

**Appendix 1**

**Test system: IEEE 33 bus distribution network**

Table 1 provides data of the line’s impedance and the load demand power at receiving end.

| **Line number** | **From bus** | **To bus** | **R (Ω)** | **X (Ω)** | **P_load_ (kW)** | **Q_load_ (kVAR)** |
| --- | --- | --- | --- | --- | --- | --- |
| **1** | **1** | **2** | **0.0922** | **0.0470** | **100** | **60** |
| **2** | **2** | **3** | **0.4930** | **0.2511** | **90** | **40** |
| **3** | **3** | **4** | **0.3660** | **0.1864** | **120** | **80** |
| **4** | **4** | **5** | **0.3811** | **0.1941** | **60** | **30** |
| **5** | **5** | **6** | **0.8190** | **0.7070** | **60** | **20** |
| **6** | **6** | **7** | **0.1872** | **0.6188** | **200** | **100** |
| **7** | **7** | **8** | **1.7114** | **1.2351** | **200** | **100** |
| **8** | **8** | **9** | **1.0300** | **0.7400** | **60** | **20** |
| **9** | **9** | **10** | **1.0440** | **0.7400** | **60** | **20** |
| **10** | **10** | **11** | **0.1966** | **0.0650** | **45** | **30** |
| **11** | **11** | **12** | **0.3744** | **0.1238** | **60** | **35** |
| **12** | **12** | **13** | **1.4680** | **1.1550** | **60** | **35** |
| **13** | **13** | **14** | **0.5416** | **0.7129** | **120** | **80** |
| **14** | **14** | **15** | **0.5910** | **0.5260** | **60** | **10** |
| **15** | **15** | **16** | **0.7463** | **0.5450** | **60** | **20** |
| **16** | **16** | **17** | **1.2890** | **1.7210** | **60** | **20** |
| **17** | **17** | **18** | **0.7320** | **0.5740** | **90** | **40** |
| **18** | **2** | **19** | **0.1640** | **0.1565** | **90** | **40** |
| **19** | **19** | **20** | **1.5042** | **1.3554** | **90** | **40** |
| **20** | **20** | **21** | **0.4095** | **0.4784** | **90** | **40** |
| **21** | **21** | **22** | **0.7089** | **0.9373** | **90** | **40** |
| **22** | **3** | **23** | **0.4512** | **0.3083** | **90** | **50** |
| **23** | **23** | **24** | **0.8980** | **0.7091** | **420** | **200** |
| **24** | **24** | **25** | **0.8960** | **0.7011** | **420** | **200** |
| **25** | **6** | **26** | **0.2030** | **0.1034** | **60** | **25** |
| **26** | **26** | **27** | **0.2842** | **0.1447** | **60** | **25** |
| **27** | **27** | **28** | **1.0590** | **0.9337** | **60** | **20** |
| **28** | **28** | **29** | **0.8042** | **0.7006** | **120** | **70** |
| **29** | **29** | **30** | **0.5075** | **0.2585** | **200** | **600** |
| **30** | **30** | **31** | **0.9744** | **0.9630** | **150** | **70** |
| **31** | **31** | **32** | **0.3105** | **0.3619** | **210** | **100** |
| **32** | **32** | **33** | **0.3410** | **0.5302** | **60** | **40** |

*The voltage at the slack bus bar number 1=12.66kV, that is represented as the base voltage for this network, while the base power is 10 MVA.

**Appendix 2**

**Test system: IEEE 69 bus distribution network**

Table 2 provides data of the line’s impedance and the load demand power at receiving end.

| **Line number** | **From bus** | **To bus** | **R (Ω)** | **X (Ω)** | **P_load_ (kW)** | **Q_load_ (kVAR)** |
| --- | --- | --- | --- | --- | --- | --- |
| **1** | **1** | **2** | **0.0005** | **0.0012** | **0** | **0** |
| **2** | **2** | **3** | **0.0005** | **0.0012** | **0** | **0** |
| **3** | **3** | **4** | **0.0015** | **0.0036** | **0** | **0** |
| **4** | **4** | **5** | **0.0251** | **0.0294** | **0** | **0** |
| **5** | **5** | **6** | **0.3660** | **0.1864** | **2.6** | **2.2** |
| **6** | **6** | **7** | **0.3810** | **0.1941** | **40.4** | **30** |
| **7** | **7** | **8** | **0.0922** | **0.0470** | **75** | **54** |
| **8** | **8** | **9** | **0.0493** | **0.0251** | **30** | **22** |
| **9** | **9** | **10** | **0.8190** | **0.2707** | **28** | **19** |
| **10** | **10** | **11** | **0.1872** | **0.0619** | **145** | **104** |
| **11** | **11** | **12** | **0.7114** | **0.2351** | **145** | **104** |
| **12** | **12** | **13** | **1.0300** | **0.3400** | **8** | **5** |
| **13** | **13** | **14** | **1.0440** | **0.3450** | **8** | **5.5** |
| **14** | **14** | **15** | **1.0580** | **0.3496** | **0** | **0** |
| **15** | **15** | **16** | **0.1966** | **0.0650** | **45.5** | **30** |
| **16** | **16** | **17** | **0.3744** | **0.1238** | **60** | **35** |
| **17** | **17** | **18** | **0.0047** | **0.0016** | **60** | **35** |
| **18** | **18** | **19** | **0.3276** | **0.1083** | **0** | **0** |
| **19** | **19** | **20** | **0.2106** | **0.0690** | **1** | **0.6** |
| **20** | **20** | **21** | **0.3416** | **0.1129** | **114** | **81** |
| **21** | **21** | **22** | **0.0140** | **0.0046** | **5** | **3.5** |
| **22** | **22** | **23** | **0.1591** | **0.0526** | **0** | **0** |
| **23** | **23** | **24** | **0.3460** | **0.1145** | **28** | **20** |
| **24** | **24** | **25** | **0.7488** | **0.2475** | **0** | **0** |
| **25** | **25** | **26** | **0.3089** | **0.1021** | **14** | **10** |
| **26** | **26** | **27** | **0.1732** | **0.0572** | **14** | **10** |
| **27** | **27** | **28** | **0.0044** | **0.0108** | **26** | **18.6** |
| **28** | **28** | **29** | **0.0640** | **0.1565** | **26** | **18.6** |
| **29** | **29** | **30** | **0.3978** | **0.1315** | **0** | **0** |
| **30** | **30** | **31** | **0.0702** | **0.0232** | **0** | **0** |
| **31** | **31** | **32** | **0.3510** | **0.1160** | **0** | **0** |
| **32** | **32** | **33** | **0.8390** | **0.2816** | **14** | **10** |
| **33** | **33** | **34** | **1.7080** | **0.5646** | **19.5** | **14** |
| **34** | **34** | **35** | **1.4740** | **0.4873** | **6** | **4** |
| **35** | **3** | **36** | **0.0044** | **0.0108** | **26** | **18.55** |
| **36** | **36** | **37** | **0.0640** | **0.1565** | **26** | **18.55** |
| **37** | **37** | **38** | **0.1053** | **0.1230** | **0** | **0** |
| **38** | **38** | **39** | **0.0304** | **0.0355** | **24** | **17** |
| **39** | **39** | **40** | **0.0018** | **0.0021** | **24** | **17** |
| **40** | **40** | **41** | **0.7283** | **0.8509** | **1.2** | **1** |
| **41** | **41** | **42** | **0.3100** | **0.3623** | **0** | **0** |
| **42** | **42** | **43** | **0.0410** | **0.0475** | **6** | **4.3** |
| **43** | **43** | **44** | **0.0092** | **0.0116** | **0** | **0** |
| **44** | **44** | **45** | **0.1089** | **0.1373** | **39.22** | **26.3** |
| **45** | **45** | **46** | **0.0009** | **0.0012** | **39.22** | **26.3** |
| **46** | **4** | **47** | **0.0034** | **0.0084** | **0** | **0** |
| **47** | **47** | **48** | **0.0851** | **0.2083** | **79** | **56.4** |
| **48** | **48** | **49** | **0.2898** | **0.7091** | **384.7** | **274.5** |
| **49** | **49** | **50** | **0.0822** | **0.2011** | **384.7** | **274.5** |
| **50** | **8** | **51** | **0.0928** | **0.0473** | **40.5** | **28.3** |
| **51** | **51** | **52** | **0.3319** | **0.1114** | **3.6** | **2.7** |
| **52** | **9** | **53** | **0.1740** | **0.0886** | **4.35** | **3.5** |
| **53** | **53** | **54** | **0.2030** | **0.1034** | **26.4** | **19** |
| **54** | **54** | **55** | **0.2842** | **0.1447** | **24** | **17.2** |
| **55** | **55** | **56** | **0.2813** | **0.1433** | **0** | **0** |
| **56** | **56** | **57** | **1.5900** | **0.5337** | **0** | **0** |
| **57** | **57** | **58** | **0.7837** | **0.2630** | **0** | **0** |
| **58** | **58** | **59** | **0.3042** | **0.1006** | **100** | **72** |
| **59** | **59** | **60** | **0.3861** | **0.1172** | **0** | **0** |
| **60** | **60** | **61** | **0.5075** | **0.2585** | **1244** | **888** |
| **61** | **61** | **62** | **0.0974** | **0.0496** | **32** | **23** |
| **62** | **62** | **63** | **0.1450** | **0.0738** | **0** | **0** |
| **63** | **63** | **64** | **0.7105** | **0.3619** | **227** | **162** |
| **64** | **64** | **65** | **1.0410** | **0.5302** | **59** | **42** |
| **65** | **11** | **66** | **0.2012** | **0.0611** | **18** | **13** |
| **66** | **66** | **67** | **0.0047** | **0.0014** | **18** | **13** |
| **67** | **12** | **68** | **0.7394** | **0.2444** | **28** | **20** |
| **68** | **68** | **69** | **0.0047** | **0.0016** | **28** | **20** |

*The voltage at the slack bus bar number 1=12.66kV, that is represented as the base voltage for this network, while the base power is 10 MVA.
